# New Homoisoflavanes, a New Alkaloid and Spirostane Steroids from the Roots of *Herreria montevidensis* Klotzsch ex Griseb. (Herreriaceae) †

**DOI:** 10.3390/molecules21111589

**Published:** 2016-11-21

**Authors:** María Dutra-Behrens, Guillermo Schmeda-Hirschmann

**Affiliations:** 1 Departamento de Produtos Naturais, Instituto de Tecnologia em Fármacos da Fundação Oswaldo Cruz–Farmanguinhos/Fiocruz, Rua Sizenando Nabuco 100, 21041-250 Rio de Janeiro, Brazil; behrens@fiocruz.br or mbehrens@far.fiocruz.br; 2 Instituto de Química de Recursos Naturales, Universidad de Talca, Casilla 747, 3460000 Talca, Chile

**Keywords:** *Herreria montevidensis* Klotzsch ex Griseb., Herreriaceae, homoisoflavanoids, flavans, spirostane steroids, alkaloid

## Abstract

The roots of the South American vine *Herreria montevidensis* Klotzsch ex Griseb. (Herreriaceae) are used in traditional medicine by several Amerindian groups of the Paraguayan Chaco. Little is known on the chemistry of the plant, despite its widespread use across the South American Chaco. From the ethyl acetate/methanol 1:1 extract of the roots, four new and one known homoisoflavanoid, two flavan derivatives, a stilbene, a new alkaloid, and three new and four known spirostane steroids were isolated. The corresponding structures were elucidated by spectroscopic and spectrometric means. The homoisoflavonoids of the plant are related to compounds isolated from the Dracaenaceae (formerly Agavaceae) sources of the Chinese crude drug Dragon’s Blood. The new alkaloid is a novel skeleton that can be used as a chemical marker of *Herreria*. The spirostane steroids suggest chemotaxonomic relations with the Liliales. This is the first comprehensive report on the chemistry of a South American *Herreria* species.

## 1. Introduction

The vine *Herreria montevidensis* Klotzsch ex Griseb. (family Herreriaceae) is abundant in the Chaco domains of South America. It is used in traditional medicine by many Amerindian groups of the Paraguayan Chaco. The Ayoreo rubbed the stem sap on the knees of children to accelerate growth [1]. Additionally, the plant is also used to treat rheumatism [1]. The Lengua-Maskoy use the mashed roots of *H. montevidensis* as an additive to mate, a traditional drink prepared with cold water [2]. The underground parts, mainly roots, are used either as water maceration or decoction as a diuretic and the stems are collected for craftwork and handicrafts [3].

Species of *Herreria* and the closely related *Smilax* are widely used in Brazilian popular medicine as a sudorific and to treat skin diseases, gout, rheumatism and syphilis [4]. The closely related species *H*. *salsaparilha* and *Smilax* spp. are known as “salsaparilla” or “salsaparrilha” and are used for the same purposes by the Paraguayan and Brazilian country-dwellers living in the Chaco and Brazilian Pantanal. Despite this widespread use, little is known on the chemistry of the South American genus *Herreria* (Herreriaceae). Isolation of gitogenin from the roots of *H. stellata* was reported [5]. According to SciFinder (accessed on 30 August 2016), a 1973 report in a local Brazilian journal informed of the occurrence of steroidal saponins in the roots of *H. montevidensis* [6], however, these saponins were neither isolated nor identified.

To gain insight into the chemistry of Paraguayan Chaco plants used by Native Americans, the objective of this investigation was the isolation and characterization of the roots constituents of *H. montevidensis*.

## 2. Results and Discussion

**Figure 1:**
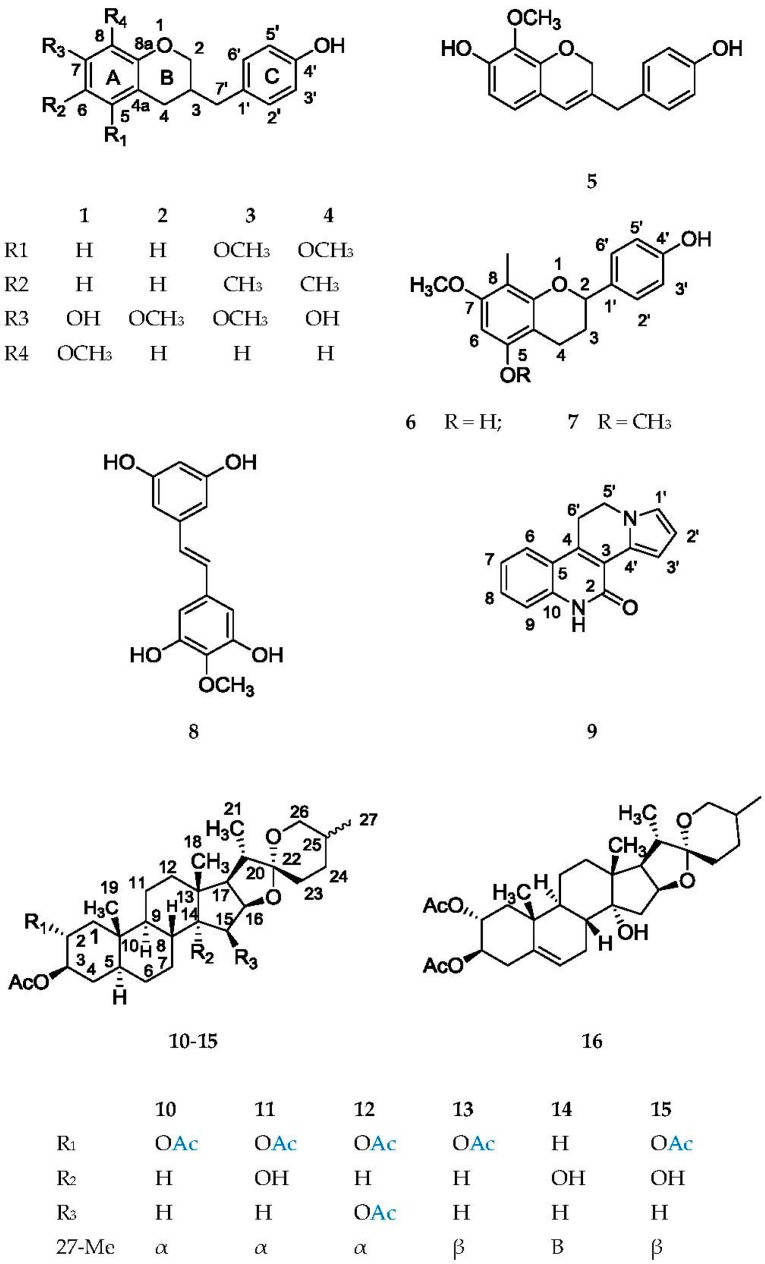
Chemical structures of the compounds **1**–**16** isolated from *Herreria montevidensis* roots.

The root extract of *Herreria montevidensis* Klotzsch ex Griseb. yielded four new (compounds **2**–**5**) and the known homoisoflavane **1**, the known flavanes **6** and **7**, the stilbene **8**, the new alkaloid **9** and seven spirostane steroids **10**–**16**, four of which (compounds **11**, **14**–**16**) are described for the first time (Figure 1).

The ^1^H-NMR spectra of compounds **1**–**4** (Table 1) showed in common a fragment composed of a central methine with three attached methylene groups (ring B), a *p*-hydroxyphenyl ring (ring C) and one to three aromatic H from a second phenyl ring (ring A), suggesting a three ring systems. The chemical shift of the methylene groups required that two of them be in benzylic positions and one bear an oxygen function. These structural elements are in agreement with the homoisoflavane skeleton. In all compounds, the B-ring was disubstituted with a hydroxy group in the *para*-position. The substitution patterns of the A-ring were deduced from the splitting of aromatic signals. The relative placement of the substituents was deduced by comparison with literature and from the NOE difference spectra. The ^13^C-NMR data of the compounds **1**–**4** clearly shows the common structural features with the t at δ 69–70 ppm for C-2, a d at δ 34–39 ppm for C-3 and two t at δ 25–30 and δ 37 ppm for C-4 and C-7′, respectively. The ^13^C-NMR data of compounds **1**–**7** is summarized in Table 2.

The ^1^H-NMR spectrum of compound **1** showed in addition to the -OCH_2_-CH-(benzylic CH_2_)_2_ sequence (ring B) and the 4-hydroxybenzyl ring (ring C), two aromatic H at δ 6.48 and δ 6.63 ppm (*J* = 8 Hz) and a methoxy s at δ 3.84 from ring A. The exact placement of the OCH_3_ was deduced from the HMBC experiments that showed clear correlations between the s peak at 3.84 ppm and the C at δ 134.8, as well as between the H-6 signal at δ 6.48 and the C at δ 134.8. The structure is in agreement with 7-hydroxy-8-methoxy-3-(4-hydroxybenzyl)chroman, previously isolated from the crude drug Dragon’s Blood [7]. The compound was also reported from *Dracaena cinnabari* [8]. The closely related compound **2** differs from **1** in the H number and sequence in the aromatic ring. The ^1^H-NMR spectrum of **2** shows three H signals at δ 6.75 (d, *J* = 8 Hz), 6.28 (dd, *J* = 8 and 2.5 Hz) and 6.21 ppm (d, *J* = 2.5 Hz), supporting a 1,2,4 sequence in the aromatic ring as well as a methoxy singlet at δ 3.77 ppm. The placement of the methoxy group at C-7 follows from the substitution pattern and shielding of H-6 and H-8 as well as from biosynthetic considerations. The molecular formula deduced from the mass spectrum (C_17_H_18_O_3_) as well as the fragmentation pattern is in agreement with the proposed structure. The compound was thus identified as 7-methoxy-3-(4-hydroxybenzyl)chroman and is reported for the first time.

The ^1^H-NMR spectra of compounds **3** and **4** (Table 1) showed only one aromatic H for the A-ring. The substituents were deduced from the typical chemical shifts as a methyl and two methoxy groups for **3** and a methyl and a methoxy group for **4**, respectively. The placement of the methoxy and methyl groups was deduced from the HMBC experiments. In the HMBC spectrum of **3**, clear correlations were observed between the methyl signal at δ 2.06 ppm, the C at δ 111.4 (C-4a) and δ 157 ppm (C-5 and C-7), indicating that the methyl group was located at C-6. Correlation experiments allowed the assignation of the methoxy signals at δ 3.66 and δ 3.75 to the C quartets at δ 59.9 and δ 55.5 ppm, respectively. The methoxy signals show clear HMBC correlations with the C at δ 157.2 and 157.3, assigned as C-8a and C-7. Further correlations were between the H singlet at δ 6.18 and the C at δ 154.0 ppm and the C at δ 154.3 and the H signals at δ 7.04 and 6.76 from the *p*-substituted aromatic ring. The ^1^H-NMR spectrum of compound **4** was close to that of **3**, showing a methyl and a methoxy signal at δ 2.08 and 3.67 ppm, respectively. The exact placement of the methyl and methoxy group follows from the HMBC experiments. Strong correlations were observed between the methyl signal at δ 2.08 and the C at δ 154.3 and δ 157.2 ppm as well as between the methoxy singlet at δ 3.67 and the C at δ 157.2. The H singlet at δ 6.13 showed clear correlation with the C at δ 152.9 ppm, while the doublets at δ 7.02 and 6.77 from the *p*-substituted aromatic ring (C) shows correlation with the C at 154.9 ppm, allowing the assignation of the oxygen-bearing aromatic C in the molecule. The HMBC correlation spectrum is shown in the Supplementary Materials. The ^13^C-NMR spectra (Table 2) are in agreement with the proposed structures. Compounds **3** and **4** were identified as 5,7-dimethoxy-6-methyl-3-(4-hydroxybenzyl)chroman and 7-hydroxy-5-methoxy-6-methyl-3-(4-hydroxybenzyl)chroman, respectively. The ^1^H-NMR, ^13^C-NMR, HSQC and HMBC spectra of compounds **1**, **3** and **4** are available in Figures S1–S12.

The ^1^H-NMR spectrum of compound **5** (Table 1) indicated identical aromatic substitution as in compound **1**. However, instead of the -OCH_2_-CH-(benzylic CH_2_)_2_ sequence observed for **1**–**4**, three br s at δ 6.04 (1H), 4.59 (2H) and 3.28 ppm (2H) indicates a double bond at C-3. This assumption was supported by the ^13^C-NMR spectrum (Table 2), showing an additional double bond (δ 128.6 s and δ 119.6 d) and the HR-EI-MS (calculated for C_17_H_16_O_4_) presenting a difference of one unsaturation degree with compound **1**. The placement of the methoxy group at C-8 was deduced by comparison with the related compound **1** as well as by the HMBC correlation of the methoxy group and the C signal at δ 134.8. The compound **5** was assigned as 7-hydroxy-8-methoxy-3-(4-hydroxybenzyl)-3-chromen and is described for the first time.

The stereochemistry at C-3 of the new homoisoflavonoids was deduced by comparison with the optical rotation of reported homoisoflavonoids. According to [8], the stereochemistry of the homoisoflavans from *Dracaena cinnabari* showed positive circular dichroism at 280 nm, indicating the same configuration for all the compounds. However, the absolute configuration by X-ray analysis was not possible [8]. The optical rotation for some of the derivatives was 0, pointing out racemic mixtures. In the report on *D. cambodiana* phenolics [9], the structure of 7,4′-dihydroxyhomoisoflavane was given without stereochemistry. In the work on Dragon’s Blood from *D. draco* [10], the compound 3-(4-dihydroxybenzyl)-5,7-dimethoxychroman was described without stereochemistry at C-3 but with optical rotation data, showing that the compound is dextrorotatory (+). The reported optical rotation for 7-hydroxy-3-(4-methoxybenzyl)chroman was (+) [11], showing a trend for this group of compounds. For the 6,4′-dihydroxy-8-methoxyhomoisoflavan, the absolute configuration at C-3 was reported as (3*R*) and the compound was dextrorotatory (+) [12]. Based on the optical rotation data and the absolute configuration reported by [12] the configuration at C-3 of the *Herreria* homoisoflavanes was assigned as (3*R*).

The structure of the compounds **6** and **7** follows the NMR spectra that show the typical signals for a flavan with a *p*-substituted B-ring and an aromatic H in the A-ring. The spectroscopic and spectrometric data of **6** and **7** are in agreement with 4′,5-dihydroxy-7-methoxy-8-methylflavan **6** [7] and 4′-hydroxy-5,7-dimethoxy-8-methylflavan **7** [13]. The ^13^C-NMR data of compounds **6** and **7** is summarized in Table 2. The flavan **6** was previously isolated from the resin of *Dracaena draco* [7] and **7** was described from *Pancratium maritimum* [13]. The compounds **6** and **7** are related to (2*S*)-5,7-dihydroxy-4′-methoxy-8-methylflavan described from *Dracaena cambodiana* [14]. The closely related compound (2*R*)-7,4′-dihydroxy-5-methoxy-8-methylflavan, differing in the relative placement of the OH and OCH_3_ functions at C-5 and C-7, was reported from *Soymida febrifuga* [12]. The compound **8**, with a molecular formula C_15_H_14_O_5_ was identified as 3,3′,5,5′-tetrahydroxy-4-methoxystilbene, previously isolated from *Phoenix dactylifera* (date palm) [15,16].

Homoisoflavonoids have been isolated previously from the red resin of the Chinese crude drug Dragon’s Blood. The resin is obtained from several botanical sources, including Palmaceae from genus *Calamus* and *Daemonorops*, Dracaenaceae (formerly Agavaceae) from genus *Dracaena*, *Pterocarpus* (Leguminosae) and *Croton* (Euphorbiaceae) species [17]. The chemistry of *Dracaena* species shows as constituents: homoisoflavonoids, flavonoid derivatives and steroids [16]. Homoisoflavonoids were reported from *D. cinnabari* [8], *D. cambodiana* [9] and *D. draco* [10]. The isolation of several stilbene derivatives and biflavonoid-like compounds was reported from *D. cochinchinensis*. The compounds showed effect against *Helicobacter pylori* and moderate thrombin inhibitory effect [17]. Homoisoflavonoids have been also isolated from the rhizomes of the Agavaceae *Agave barbadensis* [11] and from the bark of the Meliaceae *Soymida febrifuga* [12]. According to Dewick [18], related compounds isolated from *Eucomis* and *Scilla* species (Liliaceae) are biosynthetized from a chalcone-type skeleton by the addition of a carbon atom derived from methionine. Flavanes related to compound **4** with a OCH_3_ at 4′ were isolated from the rhizomes of *Agave barbadensis* [11] and from the stems of the *Dracaena cambodiana* [9]. Luo et al. [9] reported the antioxidant activity of *D. cambodiana* but the very high SC_50_ values found in the DPPH assay, compared with that or known antioxidants, indicates that they are not promising as antioxidant agents. In summary, the flavanes (homoisoflavonoids) occurring in *H. montevidensis* roots are similar to the constituents isolated from the crude drug Dragon´s Blood obtained from the *Dracaena* species.

Compound **9** was isolated as yellow needles with a HR-EI-MS of 236.0950 atomic mass units, calculated for C_15_H_12_N_2_O. The molecular formula is in agreement with an alkaloid with eleven degrees of unsaturation. A singlet at δ 172.8 ppm in the ^13^C-NMR spectrum (Table 3) indicates an α,β-unsaturated carbonyl group. Six C signals can be associated with sp^2^ C atoms belonging to an *ortho*-substituted phenyl ring while four sp^2^ C builds an additional heterocyclic five-membered ring, compatible with an α-substituted pyrrol. Two methylenes t at δ 50.4 and 25.4 ppm are part of an additional framework of the molecule. The ^1^H-NMR spectrum (Figure S13) shows three sequences including an *ortho* disubstituted aromatic ring, a α-substituted pyrrole and two vicinal methylenes. All signals in the ^13^C-NMR spectrum were assigned with the aid of 2D experiments. The HMBC spectrum was helpful for the assignment of quaternary carbons and connections of fragments. The most important long range correlations were observed between H-7, H-9, H-6′ and N-H each with C-5, supporting the 2(1*H*)-quinolinone moiety of the compound (Figure S14). HMBC correlations and NOE experiments allowed the placement of the α-substituted pyrrole and -CH_2_-CH_2_- sequence in the compound, leading to structure **9**. Among the dipolar interactions, those between H-6′ and H-6, between H-5′ and H-3′ as well as between the N-H and H-9 should be emphasized for the characterization of the compound. All information is summarized in Table 3. The new compound belongs to a novel skeleton and is named herrerin in recognition of the plant source of the compound. 

The structure of the compounds **10**–**16** was elucidated after acetylation which gave the secondary acetates. The ^1^H-NMR spectra of the compounds (Table 4) were similar, showing two angular methyl groups at δ 0.73–0.99 and δ 0.90–1.15 ppm, two methyl d at δ 0.76–1.08 and δ 0.93–1.00, two H associated with a primary alcohol/ether system (-OCH_2_-CH-) in the range δ 3.30–3.93 ppm, a deshielded H signal at 4.34–4.63 ppm and the H belonging to the acetylated hydroxy functions at δ 5.02–5.10 and δ 4.67–4.80 ppm. The ^13^C-NMR spectra (Table 5) suggest sapogenins of the spirostane type, characterized by the presence of a spiroketal ring system.

According to [19], naturally occurring spirostanes can be classified into groups according to the following structural characteristics: stereochemistry at C-5, C-22 and C-25; relative placement and stereochemistry of the functional groups and position and number of double bonds, mainly for the Δ5 (5-en) derivatives. In the IR spectra, characteristic bands for the spiroketal system can be observed at 920–930 cm^−1^ for (25*S*), stronger than at 900–905 cm^−1^ for the (25*R*) spiroketal. In the ^1^H-NMR spectrum of compounds **10**–**13** and **15**, two coupling ddd from the ester bearing centers require for each of them a vicinal CH_2_ group, supporting the sequence -CH_2_-CHOAc-CHOAc-CH_2_- and the placement of the acetates at C-2 and C-3. The *J* values of the coupling constants *J*_2_,_3_ indicates a *trans*-diaxial arrangement of the H atoms (Table 4). Additional signals at δ 4.37–4.63 and δ 3.30–3.93 are typical for H-16 and for both H-26 hydrogen atoms. The C-25 configuration can be deduced from the vicinal couplings from the geminal C-26 methylene protons (axial or equatorial position of the H in the vicinal position). For an equatorial methyl at C-25, the *J* values are *J*_25,26_ = 5 and *J*_25,26_ = 11 Hz, respectively. The ^1^H-NMR spectra of the compounds **10** and **13** were very similar, showing two acetates at H-2 and H-3 and differing in the chemical shift of the signals from H-26 (-O-CH_2_-) and the methyl d (H-27). Both compounds differ in the stereochemistry at C-25. The ^1^H- and ^13^C-NMR data of the compounds are in agreement with the 2*O*,3*O*-diacetates of gitogenin ((25*R*)-5α-spirostan-2α,3β-diol) (**10**) and neogitogenin ((25*S*)-5α-spirostan-2α,3β-diol) (**13**), respectively [19]. A clear differentiation of both compounds is possible on the basis of the ^13^C-NMR spectra. The axial methyl group at C-25 in the S-series led to a high field shift of 3–4 ppm in the α- and β-position and due to the gauche effect about 5.5 ppm on the C-23 signal. The spirostane steroid gitogenin was previously isolated from *Digitalis* spp., *Yucca gloriosa* and *Isoplexis canariensis* while neogitogenin was reported from *Digitalis*, *Yucca* and other species [16].

The ^1^H-NMR spectra of **11** and **15** were similar to each other and differ from that of **10** and **13** by the occurrence of an additional OH function in **11** and **15**. The splitting of the H-15 signal of **11** and **15** suggest that the hydroxy function is placed at C-14. The chemical shift of an additional downfield singlet at δ 87.9 ppm in the ^13^C-NMR spectra confirmed this assumption. The stereochemistry of the OH function was deduced from the chemical shift of H-17, which was shifted downfield due to the syn-planar orientation with the hydroxy group. 

Compounds **11** and **15** differ in the stereochemistry of the H-27 methyl group as can be deduced from the chemical shift of the H-26 and H-27 signals. The compounds were assigned as 14α-hydroxygitogenin ((25*R*)-5α-spirostan-2α,3β,14α-triol) (isolated as the diacetate **11**) and 14α-hydroxyneogitogenin ((25*S*)-5α-spirostan-2α,3β,14α-triol) (isolated as the diacetate **15**). Both compounds are reported for the first time as natural products. Compound **12**, with hydroxy functions at H-2 and H-3, differ from **11** in the placement of the third OH function. While compound **11** presents a tertiary alcohol at C-14, compound **12** shows the additional OH group at C-15 and was identified as digitogenin.

The ^1^H-NMR spectrum of **14** differs from the other spirostane steroids from the plant by the absence of the downfield shifted H-2 signal and the presence of only one acetate. The ^13^C-NMR data allowed the assignation of the signals by comparison with the data reported by [19] for steroidal sapogenins. The s at δ 88.1 ppm in the ^13^C-NMR spectrum indicates that the second OH function is placed at C-14. The multiplicity of the H-3 signal and the chemical shift of the H-26 protons are in agreement with 14α-hydroxy neotigogenin ((25*S*)-5α-spirostan-3α,14α-diol) (as the acetate **14**). The ^1^H-NMR spectrum of compound **16** was similar to that of compound **11**, differing by the olefinic br ddd signal at δ 5.47 ppm (*J* = 5, 2, 2 Hz), assigned to H-6, and by the ^13^C spectrum that showed a double bond at δ 137.1 (s) and 123.4 (d) ppm for compound **16**. The structural feature is typical for H-5 in steroids, supporting the Δ^5^ derivative of **11**. The ^13^C-NMR data is in agreement with the assignation. The compound was identified as 14α-hydroxy-yuccagenin ((25*R*)-spirost-5-en-2α,3β,14α-triol). The steroidal sapogenins **11**, **14**–**16** were not found in the consulted databases (SciFinder and Dictionary of Natural Products on DVD [16]) and to the best of our knowledge are reported for the first time.

The stereochemistry at C-20 from all the isolated spirostane was deduced on the basis of the NOE experiments. Clear effects were observed between H-21 and H-17 as well as between H-16 and H-17. The stereochemistry at C-22 was confirmed on the basis of the ^13^C-NMR data compared with the literature. The NOE effect between H-19 and H-2 confirm the occurrence of 5α-spirostane. Spirostane steroids, also known as sapogenins occurs in the Liliaceae, Amaryllidaceae and Dioscoreaceae families from the Monocots as well as in some Dicot families such as the Scrophulariaceae and Solanaceae. They are frequently linked with sugars to build saponins. Sapogenins are used for the commercial attainment of steroidal hormones, being diosgenin from *Dioscorea* species the most important starting product [16].

The rhizome of *Herreria montevidensis* was formerly included in the same group of sources as the crude drug “zarzaparrilla” as a *Smilax* species. Both of them have in common the traditional use as a diuretic and depurative, in Asia as well as in the Americas. Brandao et al. [20] refer to the Brazilian plants described by European naturalists in the 19th century, including “zarzaparrilla”. The common name refers to *Smilax* and *Herreria* species. A recent work on *Smilax brasiliensis* and *Herreria salsaparrilha* from Brazil showed a positive impact of the crude drugs’ extracts on the triglyceride levels in high-refined carbohydrate diets in mice [21] as well as on the glucose and cholesterol levels in treated animals. The authors detected saponins but also chlorogenic acid and known phenolics in the extracts.

Rhizomes from several *Smilax* species are used in traditional Asian medicine. Some of them contain phenylpropanoids that are active towards β-secretase [22], antioxidant and cytotoxic glycosides [23] or present anti-estrogenic/estrogenic activity [24], among other effects. Spirostane saponins also present anti-inflammatory activity [25]. The chemical diversity found in the *H. montevidensis* roots from the Paraguayan Chaco suggests the potential of the crude drug constituents as bioactive agents.

## 3. Experimental Section

### 3.1. General Experimental Procedures

Melting points were determined on a Koffler hot stage apparatus (Electrothermal 9100, Dubuque, IA, USA) and were uncorrected. IR spectra were recorded on a Nicolet Nexus 470 FT-IR instrument (Thermo Electron Corporation, Waltham, MA, USA). The NMR spectra were recorded on an Avance 400 spectrometer (Bruker, Rheinstetten, Germany) at 400 MHz for ^1^H and 100 MHz for ^13^C- in CDCl_3_ or CDCl_3_-methanol-*d*_4_. Chemical shifts are given in ppm with residual chloroform as the internal standard. High-resolution mass spectra were measured on a VG Micromass ZAB-2F at 70 eV (Varian Inc., Palo Alto, CA, USA). Merck silica gel (0.063–0.2) was used for column chromatography. Pre-coated Si gel plates (Kieselgel 60 F254, 0.25 mm, Merck, Darmstadt, Germany) were used for TLC analysis. TLC spots were visualized by spraying the chromatograms with *p*-anisaldehyde–ethanol–acetic acid–H_2_SO_4_ (2:170:20:10 *v/v*) and heating at 110 °C for 3 min.

### 3.2. Plant Material

The roots of *Herreria montevidensis* Klotzsch ex Griseb. were collected in the outskirts of the Ayoreo settlement of Isla Alta, Departamento Alto Paraguay, Paraguay, in December, 1991. Voucher herbarium specimens (Schmeda 1408) were identified by S. Smith (Smithsonian Institution, Washington, DC, USA) where they have been deposited.

### 3.3. Extraction and Isolation

The air-dried roots (750 g) were powdered and extracted with EtOAc–MeOH 1:1 (3 × 5 L) to give a crude extract which was partitioned between CHCl_3_ and H_2_O. The CHCl_3_-soluble fraction (7.5 g) was chromatographed on a medium pressure silica gel column with a petroleum ether- diethyl ether–EtOAc–MeOH gradient, to give 40 fractions of 250 mL each. The first group of fractions did not contain compounds of interest and were discarded. Fraction 1 yielded after preparative HPLC (RP8, MeOH–H_2_O 7:3), 3 mg **7** (*R*_t_ 5.4 min) and 18 mg **3** (*R*_t_ 9.1 min.). Compound **9** (11 mg) eluted in fraction 12 and recrystallized from MeOH.

Fractions 14–16 were combined and rechromatographed on SiO_2_ with a PE/EtOAc gradient to give 20 fractions. Fractions 10–14 were further purified on Sephadex LH-20 with MeOH. Fractions 12–13 from the Sephadex column yielded 7 mg **1**, while fractions 14–15 afforded after HPLC (RP8, MeOH–H_2_O 1:1) 4 mg **1** (*R*_t_ 5.6 min) and 3 mg **7** (*R*_t_ 7.3 min). Fractions 16–18 afforded after HPLC (RP8, MeOH–H_2_O 1:1) 3 mg **2** (*R*_t_ 6.3 min), 4 mg **6** (*R*_t_ 7.0 min) and 20 mg **4** (*R*_t_ 8.2 min). Fractions 19–25 afforded after HPLC (RP8, MeOH–H_2_O 1:1) 3 mg **6** (*R*_t_ 6.5 min).

Fractions 23–25 from the first silica gel column were acetylated and chromatographed on a medium pressure silica gel column with a petroleum ether (PE)/methyl *tert*-butyl ether (MTBE) gradient. Fractions 2–3 (130 mg) afforded a mixture of the acetates **10** and **13**. Some 60 mg from the fractions 2–3 yielded after preparative HPLC (PE–MTBE; 9.5:1.5) 15 mg of a **10**/**13** (3:1) mixture (*R*_t_ 11.6 min), 20 mg **10**/**13** (1:3) mixture (*R*_t_ 17.2 min) and 6 mg **13** (*R*_t_ 21.5 min). Fractions 4 and 5 afforded 186 mg **12**. Fractions 8 and 9 afforded 12 mg **14**. Fraction 11 yielded 8 mg **16**. Fraction 13 afforded 60 mg **11** and fraction 15 yielded 17 mg **15**.

The aqueous phase from the total extract partition was lyophilized and the resulting powder was extracted with MeOH. The MeOH-soluble fraction (12 g) was chromatographed on a medium pressure silica gel column with a petroleum ether-diethyl ether-EtOAc–MeOH gradient to give 50 fractions of 250 mL each. Fractions 11 and 12 yielded after HPLC (RP8, MeOH–H_2_O 7:3) 26 mg **1** and 8 mg **5**. Gel permeation of fractions 15–18 on Sephadex LH-20 with MeOH yielded 15 mg **8**. Known compounds were identified by comparing their spectral data with those of authentic material or with literature data.

### 3.4. Compound Characterization

*(3R)-7-Hydroxy-8-methoxy-3-(4-hydroxybenzyl)chroman* (**1**). Colorless resin; ^1^H-NMR and ^13^C-NMR see Table 1 and Table 2; EI-MS: *m*/*z* (rel. int.): 286 [M]^+^ (100), 178 [M − C_6_H_4_(OH)CH_3_]^+^ (40), 107 [C_7_H_7_O hydroxytropylium]^+^ (100); HR-EI-MS 286.1205 (calcd. for C_17_H_18_O_4_, 286.1205).

*(3R)-7-Methoxy-3-(4-hydroxybenzyl)chroman* (**2**). Colorless resin; ^1^H-NMR and ^13^C-NMR see Table 1 and Table 2; EI-MS *m*/*z* (rel. int.): 270 [M]^+^ (100), 162 [M − C_6_H_4_(OH)CH_3_]^+^ (40), 107 [C_7_H_7_O hydroxytropylium]^+^ (100); HR-EI-MS 270.1256 (calcd. for C_17_H_18_O_3_, 270.1256); ${\left[\alpha \right]}_{24}^{D}$ = (+)30.0 (*c* = 2 × 10^−3^ g/100 mL, MeOH).

*(3R)-5,7-Dimethoxy-6-methyl-3-(4-hydroxybenzyl)chroman* (**3**). Colorless resin; ^1^H-NMR and ^13^C-NMR see Table 1 and Table 2; EI-MS *m*/*z* (rel. int.): 314 [M]^+^ (100), 206 [M − C_6_H_4_(OH)CH_3_]^+^ (35), 107 [C_7_H_7_O hydroxytropylium]^+^ (30); HR-EI-MS 314.1518 (calcd. for C_19_H_22_O_4_, 314.1518). ${\left[\alpha \right]}_{24}^{D}$ = (+)50.7 (*c* = 15 × 10^−3^ g/100 mL, MeOH).

*(3R)-7-Hydroxy-5-methoxy-6-methyl-3-(4-hydroxybenzyl)chroman* (**4**). Colorless resin; ^1^H-NMR and ^13^C-NMR see Table 1 and Table 2; EI-MS *m*/*z* (rel. int.): 300 [M]^+^ (100), 192 [M − C_6_H_4_(OH)CH_3_]^+^ (30), 107 [C_7_H_7_O hydroxytropylium]^+^ (30); HR-EI-MS 300.1362 (calcd. for C_18_H_20_O_4_, 300.1362). ${\left[\alpha \right]}_{24}^{D}$ = (+)53.8 (*c* = 21 × 10^−3^ g/100 mL, MeOH).

*7-Hydroxy-8-methoxy-3-(4-hydroxybenzyl)-3-chromen* (**5**). Pale yellow resin; ^1^H-NMR and ^13^C-NMR see Table 1 and Table 2; EI-MS *m*/*z* (rel. int.): 284 [M]^+^ (100), 177 [M − C_6_H_4_(OH)CH_2_]^+^ (100), 107 [C_7_H_7_O hydroxytropylium]^+^ (85); HR-EI-MS 284.1059 (calcd. for C_17_H_16_O_4_, 284.1059). ${\left[\alpha \right]}_{24}^{D}$ = (+)48.5 (*c* = 8 × 10^−3^ g/100 mL, MeOH).

*Herrerin* (**9**). Yellow needles, m.p. 228 °C; ^1^H-NMR and ^13^C-NMR see Table 3; IR ν_max_ (KBr) 3307, 2936, 1580, 1571, 1473, 1442, 1357, 1311, 1251, 1163, 1115, 1049, 963, 731, 673, 549, 433 cm^−1^; EI-MS *m*/*z* (rel. int.): 236 [M]^+^ (98), 207 (14), 143 [M − C_8_H_7_N]^+^ (100); HR-EI-MS 236.0950 (calcd. for C_15_H_12_N_2_O, 236.0950).

*14α-Hydroxygitogenin ((25R)-5α-Spirostan-2α,3β,14α-triol)* (**11**) isolated as 2*O*,3*O*-diacetate. White solid, ^1^H-NMR and ^13^C-NMR see Table 4 and Table 5; IR ν_max_ (KBr) 3564, 2929, 2859, 1741, 1450, 1368, 1252, 1180, 1134, 1098, 1043, 981, 902, 869, 734, 675, 609 cm^−1^; EI-MS *m*/*z* (rel. int.): 516 [M]^+^ (12), 457 [M − OAc]^+^ (6), 444 (20), 402 (18), 387 (28), 373 (32), 149 (56), 139 [C_9_H_15_O]^+^ (100); HR-EI-MS 516.3451 (calcd. for C_31_H_48_O_6_, 516.3451).

*14α-Hydroxyneotigogenin ((25S)-5α-Spirostan-3β,14α-diol)* (**14**) isolated as 3*O*-acetate. White solid, ^1^H-NMR and ^13^C-NMR see Table 4 and Table 5; EI-MS *m*/*z* (rel. int.): 516 [M]^+^ (12), 457 [M − OAc]^+^ (6), 444 (20), 402 (18), 387 (28), 373 (32), 149 (56), 139 (C_9_H_15_O)^+^ (100); HR-EI-MS 516.3451 (calcd. for C_31_H_48_O_6_, 516.3451).

*14α-Hydroxyneogitogenin ((25S)-5α-Spirostan-2α,3β,14α-triol)* (**15**) isolated as 2*O*,3*O*-diacetate. White solid, ^1^H-NMR and ^13^C-NMR see Table 4 and Table 5; IR ν_max_ (KBr) 3542, 2941, 1743, 1729, 1449, 1371, 1307, 1232, 1178, 1133, 1059, 1001, 945, 920, 900, 866, 735, 678, 649, 605 cm^−1^; EI-MS *m*/*z* (rel. int.): 532 [M]^+^ (12), 514 [M − H_2_O]^+^ (5), 473 [M − OAc]^+^ (10), 460 (20), 418 [460 − ketene]^+^ (15), 400 [460 − AcOH]^+^ (85), 385 [400 − Me]^+^ (8), 340 [400 − AcOH]^+^ (10), 167 (35), 149 (95), 139 [C_9_H_15_O]^+^ (100); HR-EI-MS 532.3400 (calcd. for C_31_H_48_O_7_, 532.3400).

*14α-Hydroxyyuccagenin ((25R)-Spirost-5-en-2α,3β,14α-triol)* (**16**) isolated as 2*O*,3*O*-diacetate. White solid, ^1^H-NMR and ^13^C-NMR see Table 4 and Table 5; IR ν_max_ (KBr) 3550, 2951, 2872, 1738, 1455, 1369, 1239, 1180, 1101, 1053, 980, 919, 900, 869, 732, 648, 606 cm^−1^. EI-MS *m*/*z* (rel. int.): 530 [M]^+^ (1), 512 [M − H_2_O]^+^ (1), 452 [512 − AcOH]^+^ (4), 392 [452 − AcOH]^+^ (12), 377 [392 − Me]^+^ (45), 177 (68), 139 [C_9_H_15_O]^+^ (50), 43 (100); HR-EI-MS 530.3244 (calcd. for C_31_H_46_O_7_, 530.3244).

## 4. Conclusions

Several new compounds, including the rare homoisoflavanes **2**–**5**, a new alkaloid **9** belonging to a novel skeleton type and three new spirostane steroids **11**, **15** and **16** were isolated and identified from the roots of *H. montevidensis*. The findings show clear chemotaxonomic relations with the Liliaceae and Agavaceae plant family, but with distinctive compounds that up to this point have not been identified in other species. To confirm the possible effect of the crude drug in traditional medicine, additional studies are needed, including bioactivity testing using suitable bioassays and chemical profiling of the extracts using hyphenated techniques.

## Figures and Tables

**Table 1:** ^1^H-NMR-Spectral Data of Compounds **1**–**5** (400 MHz, CDCl_3_, or CDCl_3_ + MeOH-*d*_4_, δ-values in ppm, *J* values in Hz).

Position	1 CDCl_3_ + MeOH-*d*_4_	2	3 CDCl_3_ + MeOH-*d*_4_	4 CDCl_3_ + MeOH-*d*_4_	5
2_1_	4.24 dd (10.4, 1.2)	4.06 dd (10.4, 1.2)	4.09 dd (10.4, 1.2)	4.07 dd (10.4, 1.2)	4.59 brs
2_2_	3.83 dd (10.4, 8.8)	3.69 dd (10.4, 8.8)	3.77 dd (10.4, 8.8)	3.73 dd (10.4, 8.8)	-
3	2.25 m	2.15 m	2.20 m	2.17 m	-
4_1_	2.73 dd (16.0, 5.2)	2.62 dd (16.0, 5.2)	2.79 dd (16.0, 5.2)	2.78 dd (16.0, 5.2)	6.04 brs
4_2_	2.41 dd (16.0, 8.8)	2.33 dd (16.0, 8.0)	2.37 dd (16.0, 8.0)	2.35 dd (16.0, 8.4)	
5	6.63 d (8.4)	6.75 d (8.4)	-	-	6.53 d (8.0)
6	6.48 d (8.4)	6.28 dd (8.4, 2.5)	-	-	6.44 d (8.0)
8	-	6.21 d (2.5)	6.18 s	6.13 s	-
2′,6′	7.04 d (7.6)	6.95 d (8.0)	7.04 d (8.0)	7.02 d (8.0)	7.01 d (8.0)
3′,5′	6.78 d (7.6)	6.70 d (8.0)	6.75 d (8.0)	6.77 d (8.0)	6.73 d (8.0)
7′_1_	2.58 dd (13.6, 7.6)	2.52 dd (15.2, 7.6)	2.69 dd (15.2, 7.6)	2.61 dd (13.6, 7.2)	3.28 brs
7′_2_	2.52 dd (13.6, 7.2)	2.45 dd (15.2, 7.6)	2.59 dd (15.2, 7.6)	2.56 dd (13.6, 7.2)	
Me	-	-	2.06 s	2.08 s	-
OMe	3.84 s	3.77 s	3.66 s	-	3.81 s
OMe	-	-	3.75 s	3.67 s	-

**Table 2:** ^13^C-NMR Spectral Data of Compounds **1**–**7** (100 MHz, CDCl_3_ or CDCl_3_ + MeOH-*d*_4_, δ-values in ppm).

Position	1 CDCl_3_ + MeOH-*d*_4_	2	3 CDCl_3_ + MeOH-*d*_4_	4 CDCl_3_ + MeOH-*d*_4_	5	6	7
2	70.0 t	69.9 t	69.6 t	69.6 t	68.0 t	77.1 d	77.0 d
3	34.0 d	34.3 d	33.9 d	33.9 d	128.6 s	19.4 t	19.5 t
4	30.3 t	30.2 t	25.5 t	25.5 t	119.6 d	29.4 t	29.5 t
4 a	114.4 s	112.8 s	111.4 s	110.0 s	116.6 s	103.9 s	106.2 s
5	124.4 d	124.4 d	157.3 s *	157.2 s	121.1 d	155.7 s	155.7 s
6	107.5 d	108.1 d	107.0 s	106.5 s	107.6 d	90.9 d	87.8 d
7	147.5 s	150.0 s	154.0 s	154.3 s	149.0 s	154.1 s	155.0 s
8	134.8 s	102.8 d	95.3 d	98.7 d	134.8 s	102.6 s	103.5 s
8a	147.2 s	154.8 s	157.2 s *	152.9 s	145.2 s	152.9 s	155.0 s
1′	130.4 s	130.3 s	131.6 s	130.5 s	131.0 s	133.5 s	134.4 s
2′, 6′	129.9 d	129.9 d	130.1 d	129.8 d	129.8 d	127.1 d	127.2 d
4‘	154.8 s	154.9 s	154.3 s	154.9 s	155.2 s	156.0 s	156.6 s
3′, 5′	115.2 d	115.2 d	115.2 d	115.1 d	115.2 d	115.1 d	115.1 d
7′	36.9 t	37.0 t	37.2 t	37.2 t	38.8 t	-	-
CH_3_	-	-	8.5 q	8.2 q	-	7.6 q	7.8 q
5-OCH_3_	-	-	59.9 q	59.7 q	-	-	56.0 q **
7-OCH_3_	-	60.7 q	55.5 q	-	-	55.3 q	55.5 q **
8-OCH_3_	60.7 q	-	-	-	60.8 q	-	-

* and **: assignations can be reversed.

**Table 3:** NMR Spectral Data of Compound **9** (400 MHz for ^1^H- and 100 MHz for ^13^C-, CDCl_3_, δ-values in ppm, *J* in Hz).

Position	δ_C_, Type	H	δ_H_ (*J* in Hz)	HMBC	NOE
2	172.8 C				
3	132.7 C				
4	121.0 C				
5	127.2 C				
6	120.6 CH	6	7.66 d (8)	4 (w), 5 (w), 8 (s), 10 (s)	6′(3)
7	120.3 CH	7	7.17 ddd (8, 8, 1)	5 (s), 9 (s)	
8	126.3 CH	8	7.36 ddd (8, 8, 1)	6 (s), 10 (s)	
9	112.3 CH	9	7.46 d (8)	5 (s), 7 (s)	N-H(3)
10	136.6 C				
1′	119.9 CH	1′	7.34 dd (4, 2)	3′ (m), 4′ (m)	
2′	109.3 CH	2′	6.26 dd (4, 2)	1′ (w), 3′ (s), 4′ (s)	
3′	129.6 CH	3′	6.90 dd (2, 2)	1′ (s), 2′ (s), 5′ (w), 4′ (s)	5´(3)
4′	133.9 C	-			
5′	50.4 CH_2_	5′	4.48 m	3′ (m), 6′ (m), 4′ (m), 4 (s)	3′(5)
6′	25.4 CH_2_	6′	3.40 m	3 (s), 4 (s), 5 (s), 5′ (m)	6 (5)
NH			9.45 br s	5 (w)	9 (5)

s: strong; m: medium; w: weak interaction.

**Table 4:** Selected ^1^H-NMR Spectral Data of Compounds **10**–**16** (400 MHz, CDCl_3_, δ-values in ppm, *J* in Hz).

Position	10	11	12	13	14	15	16
2	5.02 ddd (12, 10, 5)	5.03 ddd (12, 10, 5)	5.04 ddd (12, 10, 5)	5.02 ddd (12, 10, 5)	^‡^	5.03 ddd (12, 10, 5)	5.10 ddd (12, 10, 5)
3	4.78 ddd (11, 10, 5)	4.79 ddd (11, 10, 5)	4.80 ddd (11, 10, 5)	4.78 ddd (11, 10, 5)	4.67 dddd (11, 11, 4, 4)	4.79 ddd (11, 10, 5)	4.72 ddd (11, 10, 5)
6	*	*	*	*	*	*	5.47 br ddd (5,2,2)
15 α	1.65 m	1.94 dd (13, 8)	4.10 ddd (5, 3, 2)	1.65 m	1.94 dd (13, 8)	1.94 dd (13, 8)	^‡^
15 β	1.20 m	1.59 dd (13, 6)	15-OH, 2.23 brs	1.20 m	1.58 dd (13, 6)	1.59 dd (13, 6)	^‡^
16	4.37 ddd (8, 8, 6)	4.62 ddd (8, 8, 6)	4.34 dd (8, 5)	4.37 ddd (8, 8, 6)	4.63 ddd (8, 8, 6)	4.62 ddd (8, 8, 6)	4.62 ddd (8, 8, 6)
17	1.75 dd (8, 7)	2.31 dd (8, 7)	1.94 dd (8, 7)	1.75 dd (8, 7)	2.31 dd (8, 7)	2.31 dd (8, 7)	2.32 dd (8, 7)
18	0.73 s	0.90 s	0.99 s	0.73 s	0.86 s	0.90 s	0.93 s
19	0.90 s	0.95 s	0.95 s	0.90 s	0.91 s	0.95 s	1.15 s
20	1.85 m	1.90 m	^‡^	1.80 m	1.86 m	1.86 m	^‡^
21	0.93 d (7)	0.97 d (7)	0.96 d (7)	0.96 d (7)	1.00 d (7)	1.00 d (7)	0.99 d (7)
26 α	3.34 dd (11, 11)	3.36 dd (11, 11)	3.38 dd (11, 11)	3.27 brd (11)	3.30 brd (11)	3.30 brd (11)	3.36 dd (11, 11)
26 β	3.45 ddd (11, 5, 2)	3.48 ddd (11, 5, 2)	3.51 ddd (11, 5, 2)	3.92 dd (11, 3)	3.93 dd (11, 3)	3.93 dd (11, 3)	3.48 ddd (11, 5, 2)
27	0.76 d (7)	0.79 d (7)	0.80 d (7)	1.05 d (7)	1.08 d (7)	1.08 d (7)	0.79 d (7)
OAc	2.01 s	2.01 s	2.01 s	2.01 s	2.01 s	2.01 s	2.01 s
	2.00 s	2.01 s	2.00 s	2.00 s	-	2.01 s	2.00 s

* overlapped multiplet; ^‡^ not estimated.

**Table 5:** ^13^C-NMR Spectral Data of Compounds **10**–**16** (100 MHz, CDCl_3_, δ-values in ppm).

Position	10	11	12	13	14	15	16
1	42.1 t	42.4 t	42.1 t	42.3 t	36.8 t	42.3 t	42.3 t
2	71.6 d	71.8 d	71.8 d	71.9 d	28.3 t	71.8 d	74.3 d
3	74.4 d	74.5 d	74.5 d	74.6 d	73.5 d	74.5 d	71.4 d
4	32.5 t	32.8 t	32.6 t	32.7 t	33.9 t	32.7 t	36.2 t
5	43.9 d	43.9 d	44.2 d	44.1 d	44.3 d	43.8 d	137.1 s
6	27.4 t	27.4 t	27.4 t	27.5 t	27.4 t	27.4 t	123.4 d
7	31.5 t	26.7 t	31.1 t	31.6 t	26.9 t	26.7 t	26.0 t
8	34.2 d	37.8 d	30.5 d	34.3 d	38.4 d	37.7 d	34.3 d
9	53.8 d	46.6 d	54.5 d	54.0 d	46.7 d	46.6 d	43.0 d
10	36.9 s	37.3 s	37.2 s	37.1 s	35.8 s	37.3 s	38.3 s
11	21.0 t	20.1 t	21.3 t	21.1 t	20.0 t	20.1 t	19.7 t
12	39.6 t	31.8 t	42.1 t	39.8 t	31.9 t	31.8 t	31.4 t
13	40.3 s	44.5 s	40.6 s	40.4 s	44.5 s	44.5 s	44.3 s
14	55.8 d	87.9 s	60.2 d	56.0 d	88.1 s	87.9 s	87.1 s
15	31.7 t	39.5 t	69.6 d	31.8 t	39.3 t	39.4 t	39.3 s
16	80.6 d	80.8 d	82.0 d	80.8 d	81.0 d	80.9 d	80.7 d
17	61.7 d	58.7 d	61.1 d	61.9 d	58.5 d	58.5 d	58.8 d
18	16.3 q	20.1 q	19.0 q	16.4 q	20.2 q	20.1 q	19.8 q
19	12.8 q	12.8 q	12.8 q	12.9 q	12.1 q	12.8 q	14.7 q
20	41.4 d	41.6 d	42.4 s	42.1 d	42.1 d	42.1 d	41.6 d
21	14.2 q	14.7 q	14.2 q	14.3 q	14.5 q	14.5 q	14.7 q
22	109.0 s	109.5 s	110.0 s	109.6 s	109.9 s	109.9 s	109.5 s
23	31.2 t	31.5 t	31.1 t	25.7 t	25.8 t	25.8 t	31.4 t
24	28.6 t	28.8 t	28.5 t	25.9 t	26.0 t	26.0 t	28.8 t
25	30.1 d	30.2 d	30.1 d	27.0 d	27.0 d	27.0 d	30.2 d
26	66.6 t	66.8 t	67.1 t	65.1 t	65.1 t	65.1 t	66.8 t
27	17.0 q	17.1 q	17.0 q	16.0 q	16.1 q	16.1 q	17.1 d
OAc	170.3 s	170.6 s	170.5 s	170.6 s	170.7 s	170.6 s	170.5 s
	21.0 q	21.1 q	21.1 q	21.1 q	21.5 q	21.1 q	21.2 q
OAc	170.2 s	170.6 s	170.4 s	170.5 s	-	170.6 s	170.4 s
	21.0 q	21.2 q	21.1 q	21.1 q	-	21.2 q	21.1 q

## Supplementary Materials

The following are available online at www.mdpi.com/1420-3049/21/11/1589/s1.

## References

[1] Schmeda-Hirschmann G. (1993). Magic and medicinal plants of the Ayoreos of the Chaco Boreal. J. Ethnopharmacol..

[2] Arenas P. (1981). Etnobotánica Lengua-Maskoy.

[3] Alvarez J.M., Lopes R.C., Bortolotto I.M. (2008). The ethnobotany of *Herreria montevidensis* Klotzsch *ex* Griseb.-Herreriaceae, in Corumbá, Brazil. Econ. Bot..

[4] Correia P. (1984). Dicionário das Plantas úteis do Brasil.

[5] Hegnauer R. (1963). Chemotaxonomie der Pflanzen.

[6] Cecy C., Yassumoto Y. (1973). Steroidal saponins in the roots of *Herreria montevidensis* (Liliaceae). Trib. Farm..

[7] Camarda L., Merlini L., Nasini G. (1983). Dragon’s blood from *Dracaena draco*, structure of novel homoisoflavonoids. Heterocycles.

[8] Masaoud M., Ripperger H., Porzel A., Adam G. (1995). Flavonoids of Dragon’s Blood from *Dracaena cinnabari*. Phytochemistry.

[9] Luo Y., Wang H., Xu X., Mei W., Dai H. (2010). Antioxidant phenolic compounds of *Dracaena cambodiana*. Molecules.

[10] González G.A., León F., Sánchez-Pinto L., Padrón J.I., Bermejo J. (2000). Phenolic compounds of Dragon’s Blood from *Dracaena draco*. J. Nat. Prod..

[11] Tinto W.F., Simmons-Boyce J.L., McLean S., Reynolds W.F. (2005). Constituents of *Agave americana* and *Agave barbadensis*. Fitoterapia.

[12] Awale S., Miyamoto T., Linn T.Z., Li F., Win N.N., Tezuka Y., Esumi H., Kadota S. (2009). Cytotoxic constituents of *Soymida febrifuga* from Myanmar. J. Nat. Prod..

[13] Ali A.A., Makboul M.A., Attia A.A., Ali D.T. (1990). Chromones and flavans from *Pancratium maritimum*. Phytochemistry.

[14] Liu J., Dai H.-F., Wu J., Zeng Y.-B., Mei W.-L. (2008). Flavanes from *Dracaena cambodiana*. Z. Naturforsch..

[15] Fernández M.I., Pedro J.R., Seoane E. (1983). Two polyhydroxystilbenes from stems of *Phoenix dactylifera*. Phytochemistry.

[16] Buckingham J. (2016). Dictionary of Natural Products on DVD, Version 25:1.

[17] Zhu Y., Zhang P., Yu H., Li J., Wang M.-W., Zhao W. (2007). Anti-*Helicobacter pylori* and thrombin inhibitory components from Chinese Dragon’s Blood, *Dracaena cochinchinensis*. J. Nat. Prod..

[18] Dewick P.M. (1975). Biosynthesis of the 3-benzylchroman-4-one eucomin in *Eucomis bicolor*. Phytochemistry.

[19] Agrawal P.K., Jain D.C., Gupta R.K., Thakur R.S. (1985). Carbon-13 NMR spectroscopy of steroidal sapogenins and steroidal saponins. Phytochemistry.

[20] Brandão M.G.L., Zanetti N.N.S., Oliveira P., Grael C.F.F., Santos A.C.P., Monte-Mór R.L.M. (2008). Brazilian medicinal plants described by 19th century European naturalists and in the Official Pharmacopoeia. J. Ethnopharmacol..

[21] Pereira F.L., Oliveira V.B., Viana C.T.R., Campos P.P., Silva M.A.N., Brandão M.G.L. (2015). Antihyperlipidemic and antihyperglycemic effects of the Brazilian salsaparrilhas *Smilax brasiliensis* Spreng. (Smilacaceae) and *Herreria salsaparrilha* Mart. (Agavaceae) in mice treated with a high-refined-carbohydrate containing diet. Food Res. Int..

[22] Jeon S.-Y., Kwon S.-H., Seong Y.-H., Bae K., Hur J.-M., Lee Y.-Y., Suh D.-Y., Song K.-S. (2007). β-Secretase (BACE1)-inhibiting stilbenoids from Smilax Rhizoma. Phytomedicine.

[23] Wang W.-X., Li T.-X., Ma H., Zhang J.-F., Jia A.-Q. (2013). Tumoral cytotoxic and antioxidative phenylpropanoid glycosides in *Smilax riparia* A. DC. J. Ethnopharmacol..

[24] Wungsintaweekul B., Umehara K., Miyase T., Noguchi H. (2011). Estrogenic and anti-estrogenic compounds from the Thai medicinal plant, *Smilax corbularia* (Smilacaceae). Phytochemistry.

[25] Wang Y., Li C., Xiang L., Huang W., He X. (2016). Spirostane saponins from Chinese onion (*Allium chinense*) exert pronounced anti-inflammatory and anti-proliferative activities. J. Funct. Foods.

