# Supplementary material for: New Homoisoflavanes, a New Alkaloid and Spirostane Steroids from the Roots of Herreria montevidensis Klotzsch ex Griseb. (Herreriaceae)
Source: Molecules. 2016 Nov 21;21(11):1589. doi: 10.3390/molecules21111589 (PMC6274347; doi:10.3390/molecules21111589)
Supplement: Supplementary file 1 [file molecules-21-01589-s001.pdf]

Supplementary Materials: New Homoisoflavanes, a New Alkaloid and Spirostane Steroids from the Roots of *Herreria montevidensis* Klotzsch ex Griseb. (Herreriaceae)

María Dutra-Behrens and Guillermo Schmeda-Hirschmann

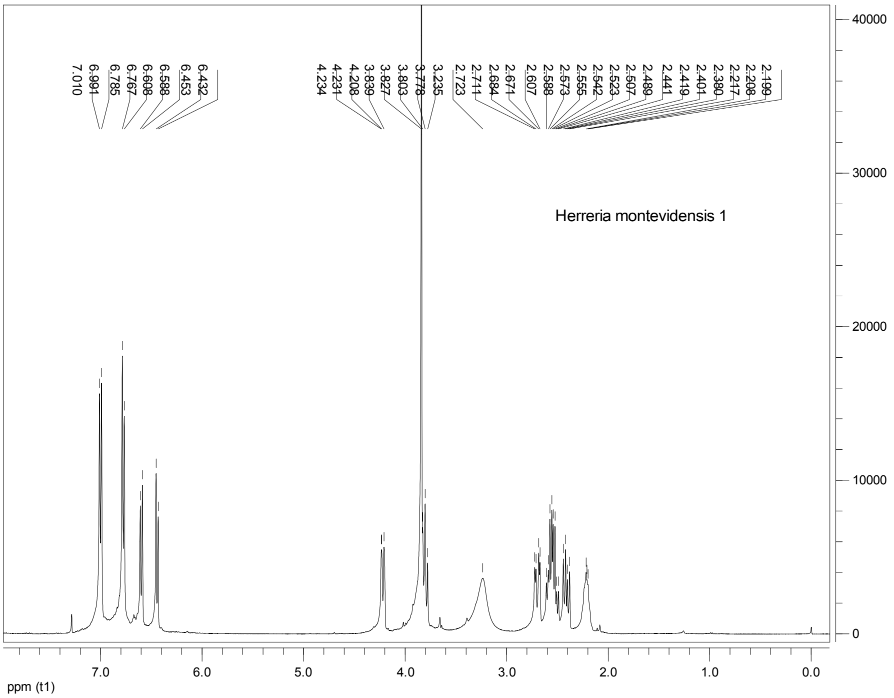

Figure S1. ^{1}H-NMR (400 MHz, $CDCl_3$-MeOH-*d*$_4$) spectrum of compound **1**.

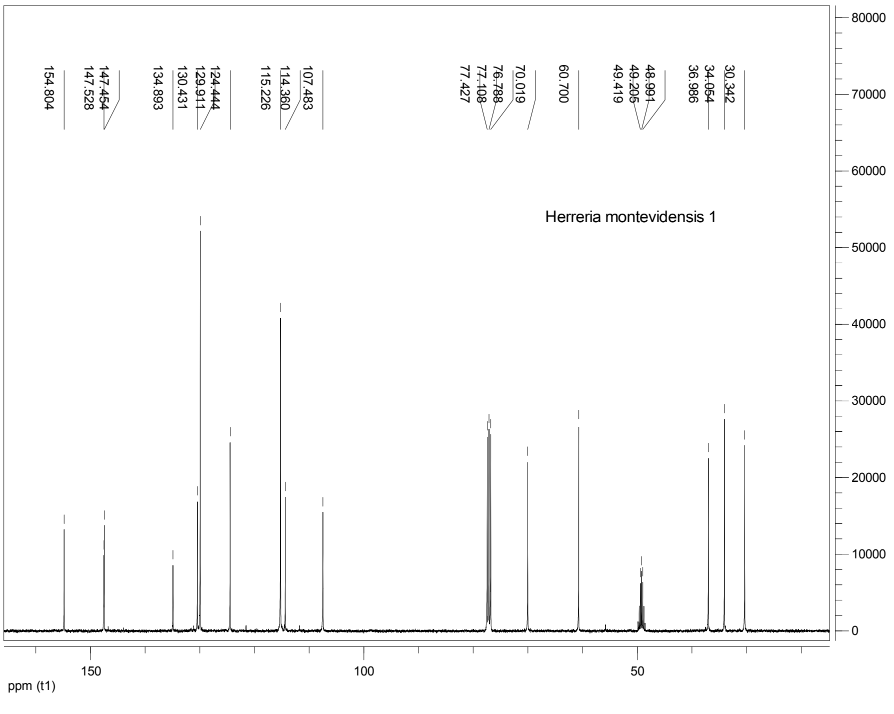

Figure S2. ^{13}C-NMR (100 MHz, $CDCl_3$-MeOH-*d*$_4$) spectrum of compound **1**.

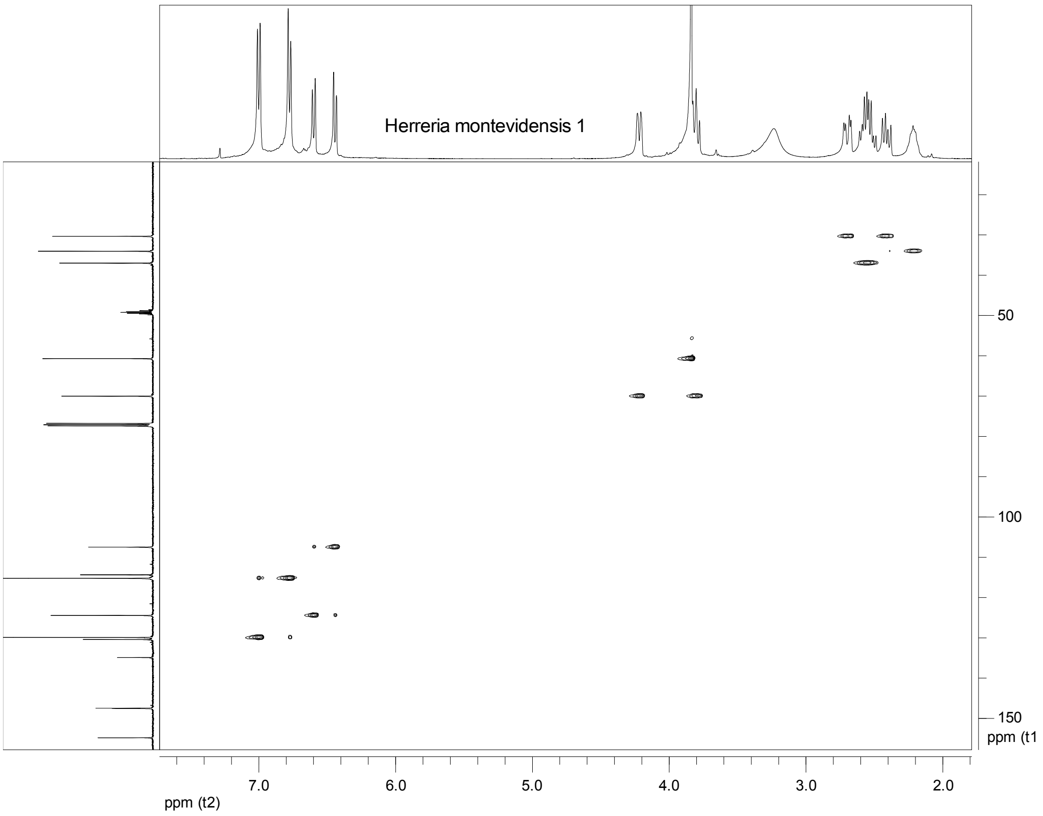

Figure S3. HSQC spectrum of compound **1**.

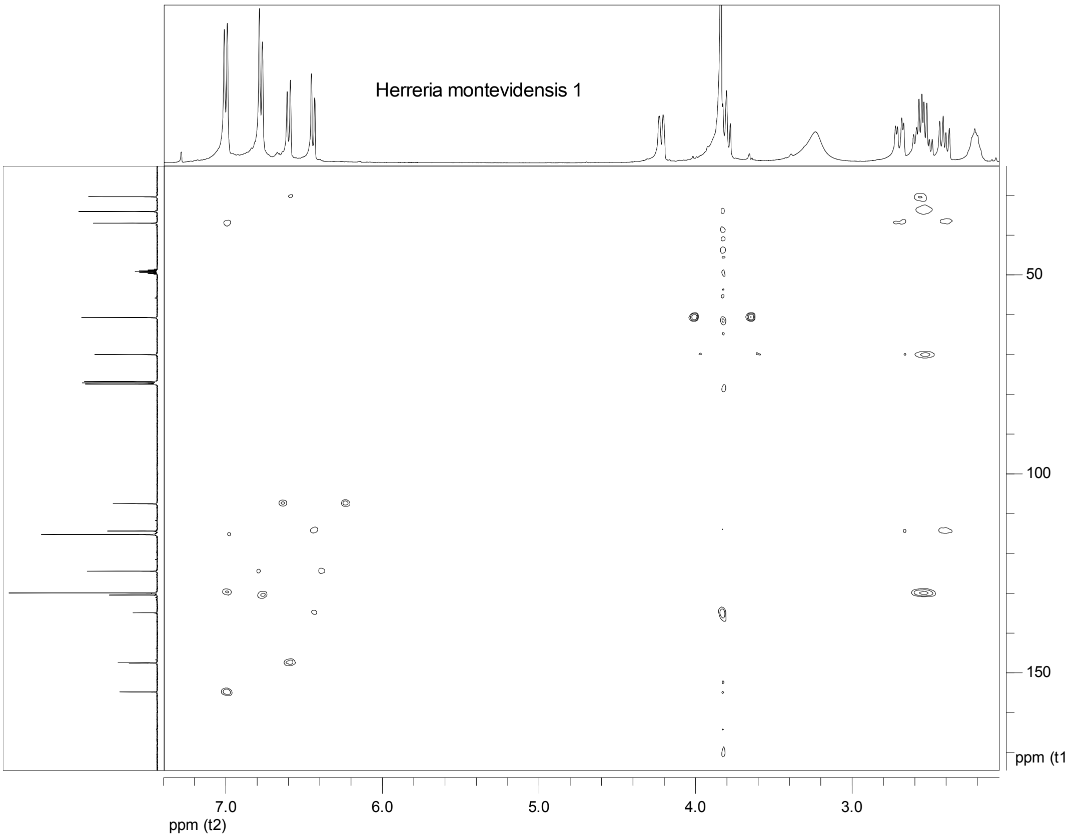

Figure S4. HMBC spectrum of compound **1**.

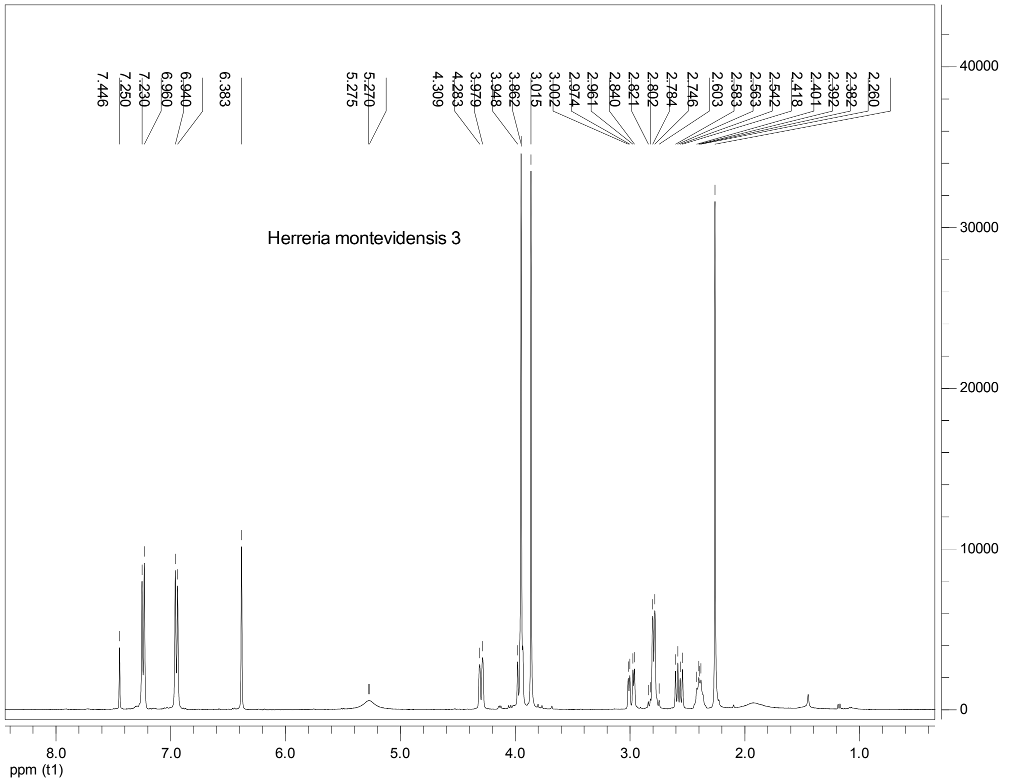

Figure S5. ^{1}H-NMR (400 MHz, $CDCl_3$-MeOH-d_4) spectrum of compound **3**.

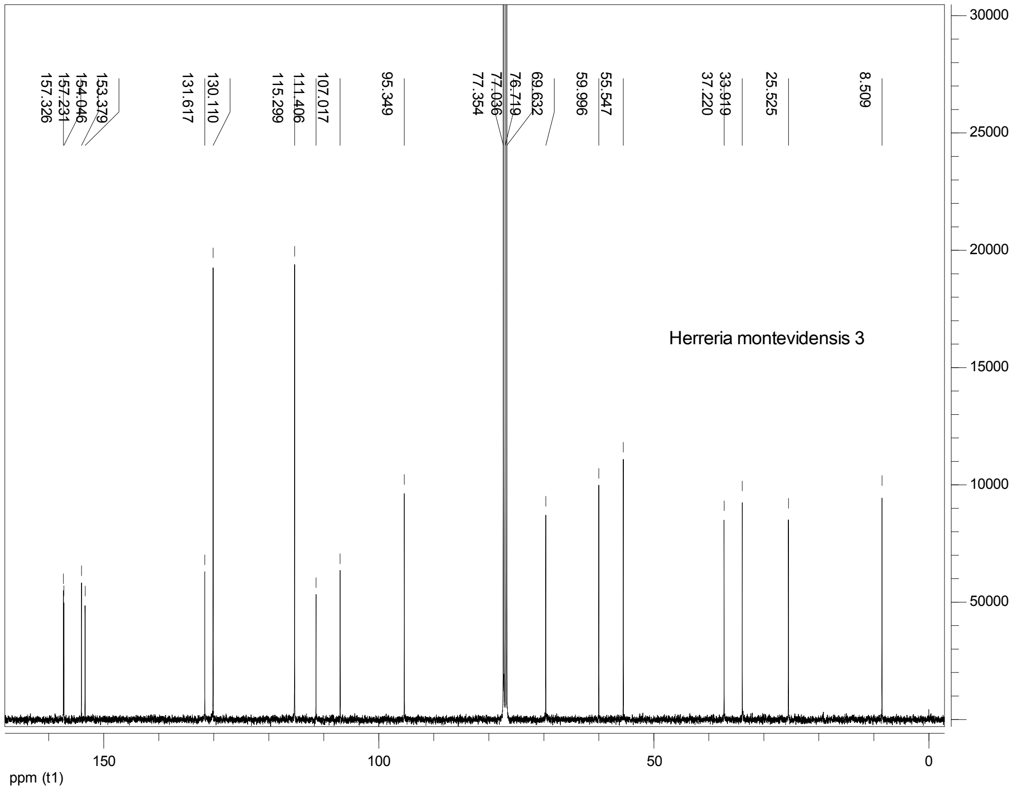

Figure S6. ^{13}C-NMR (100 MHz, $CDCl_3$-MeOH-d_4) spectrum of compound **3**.

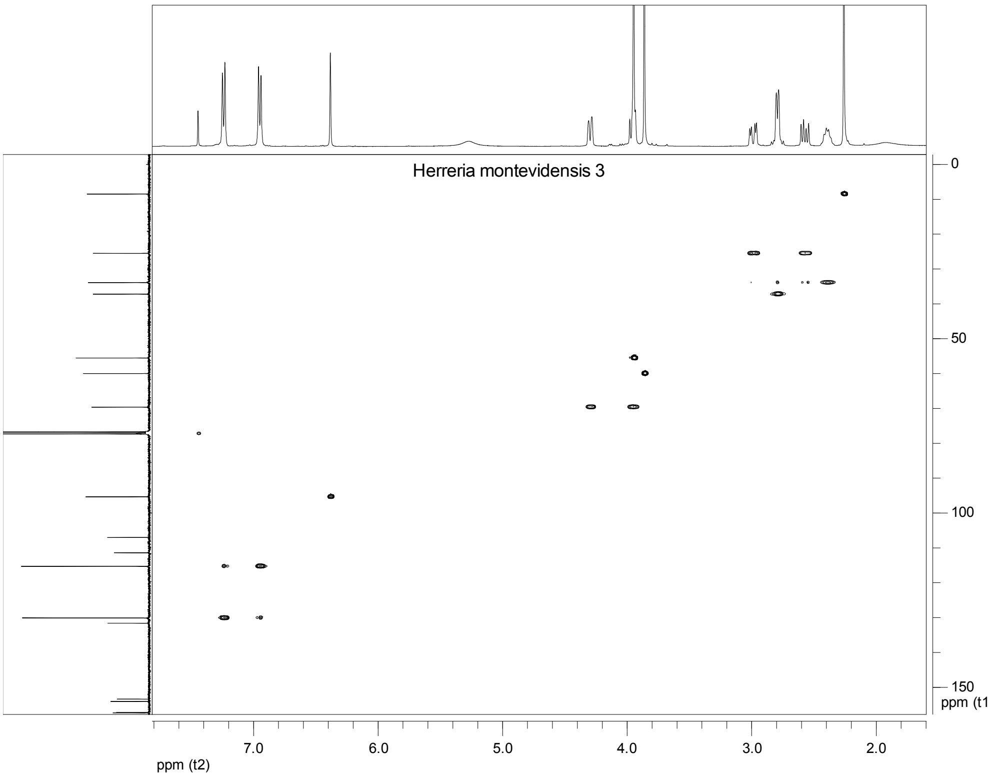

Figure S7. HSQC spectrum of compound **3**.

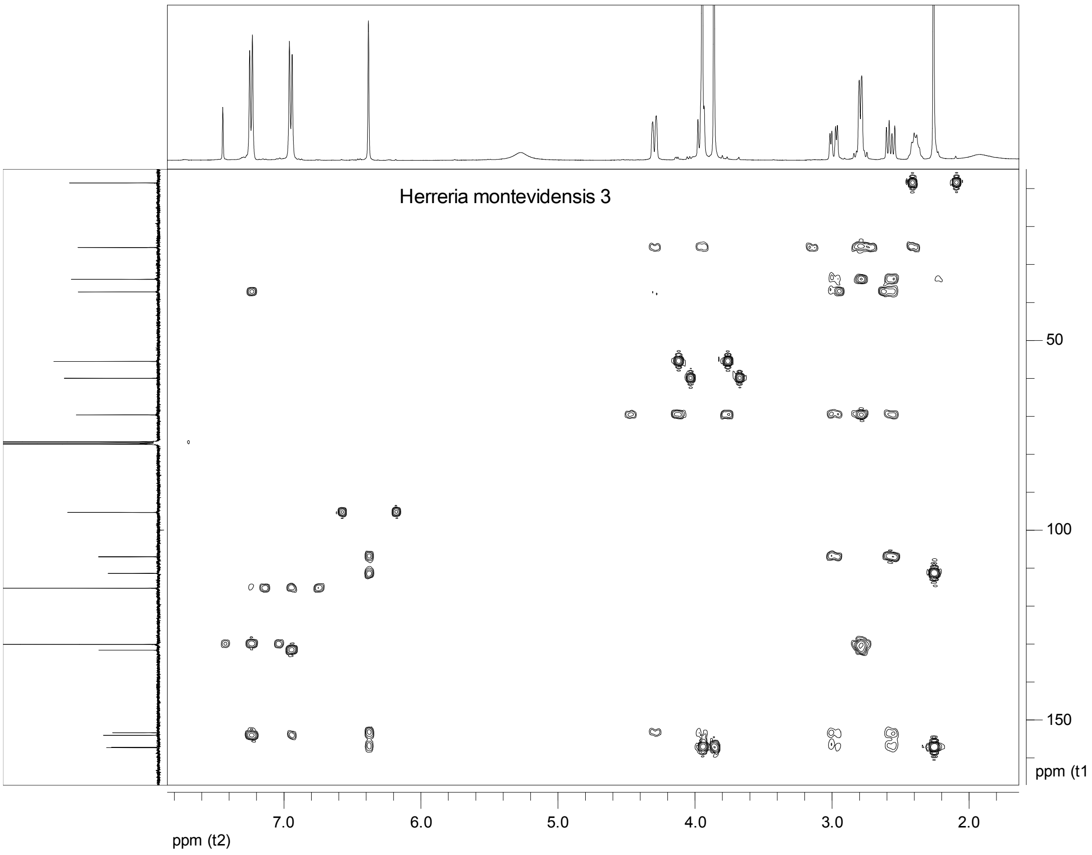

Figure S8. HMBC spectrum of compound **3**.

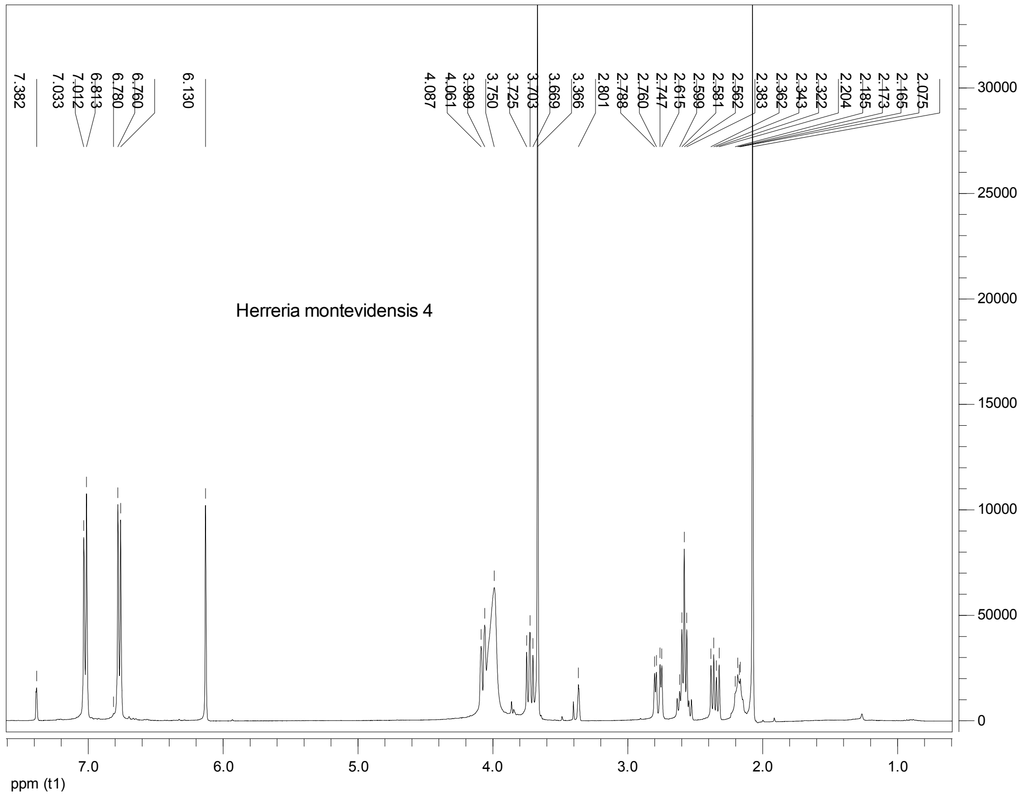

Figure S9. ^{1}H-NMR (400 MHz, $CDCl_3$-MeOH-d_4) spectrum of compound **4**.

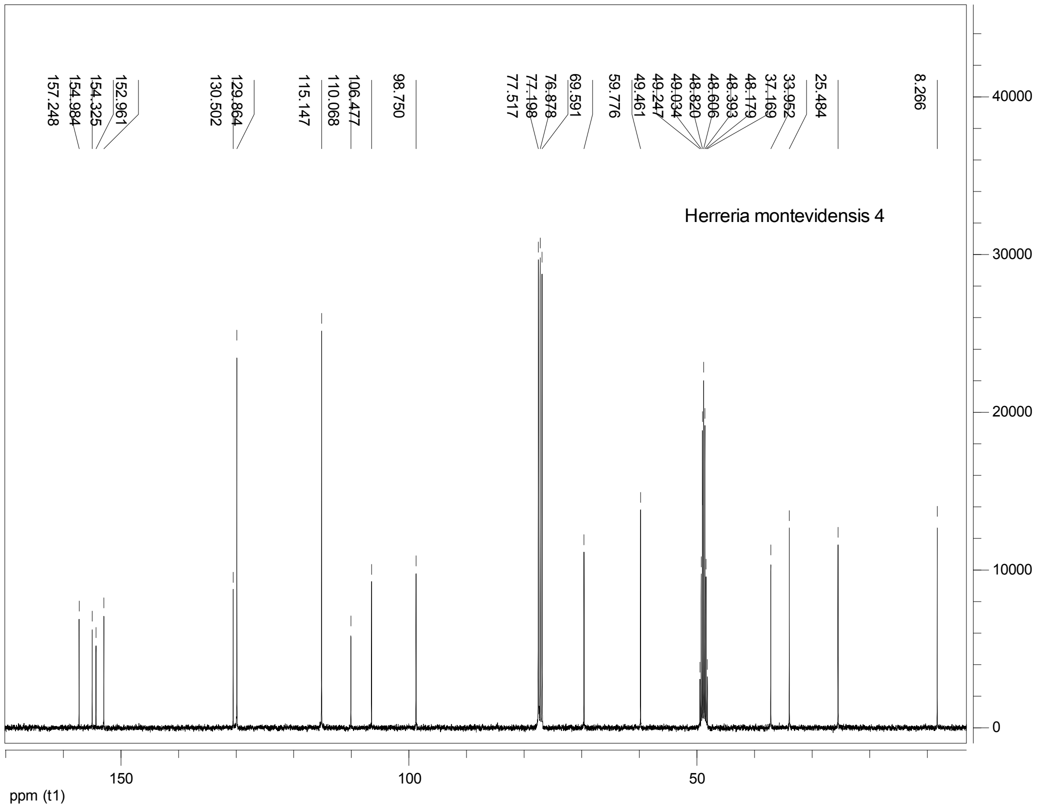

Figure S10. ^{13}C-NMR (100 MHz, $CDCl_3$-MeOH-d_4) spectrum of compound **4**.

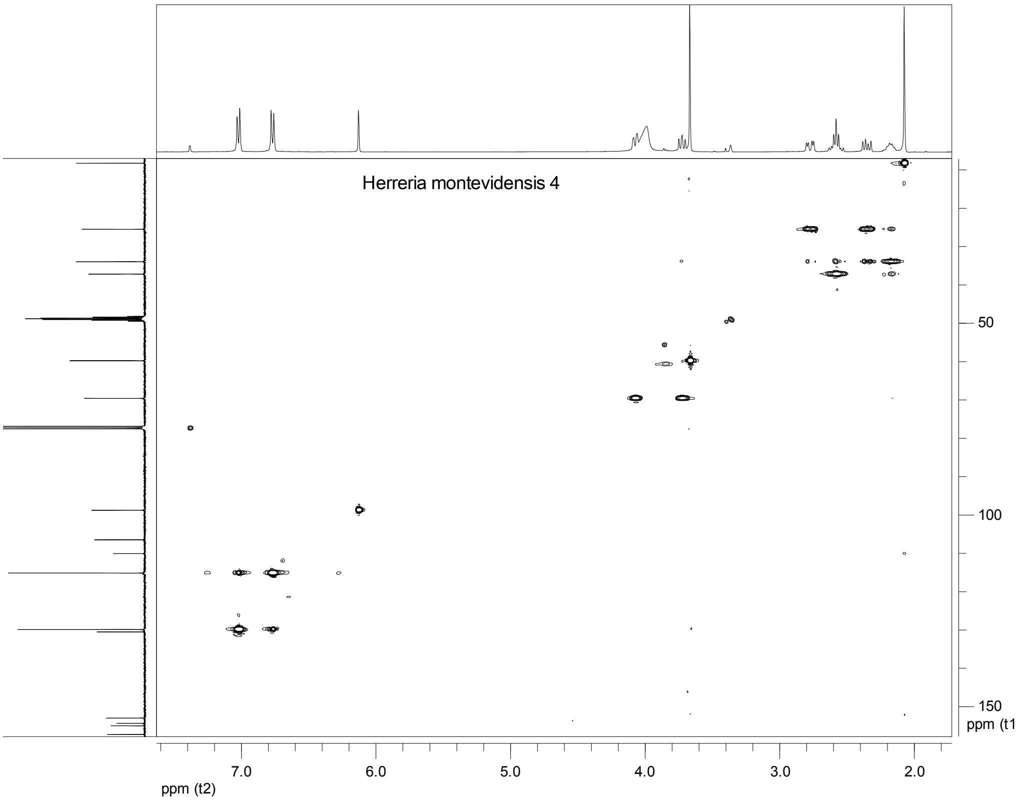

Figure S11. HSQC spectrum of compound **4**.

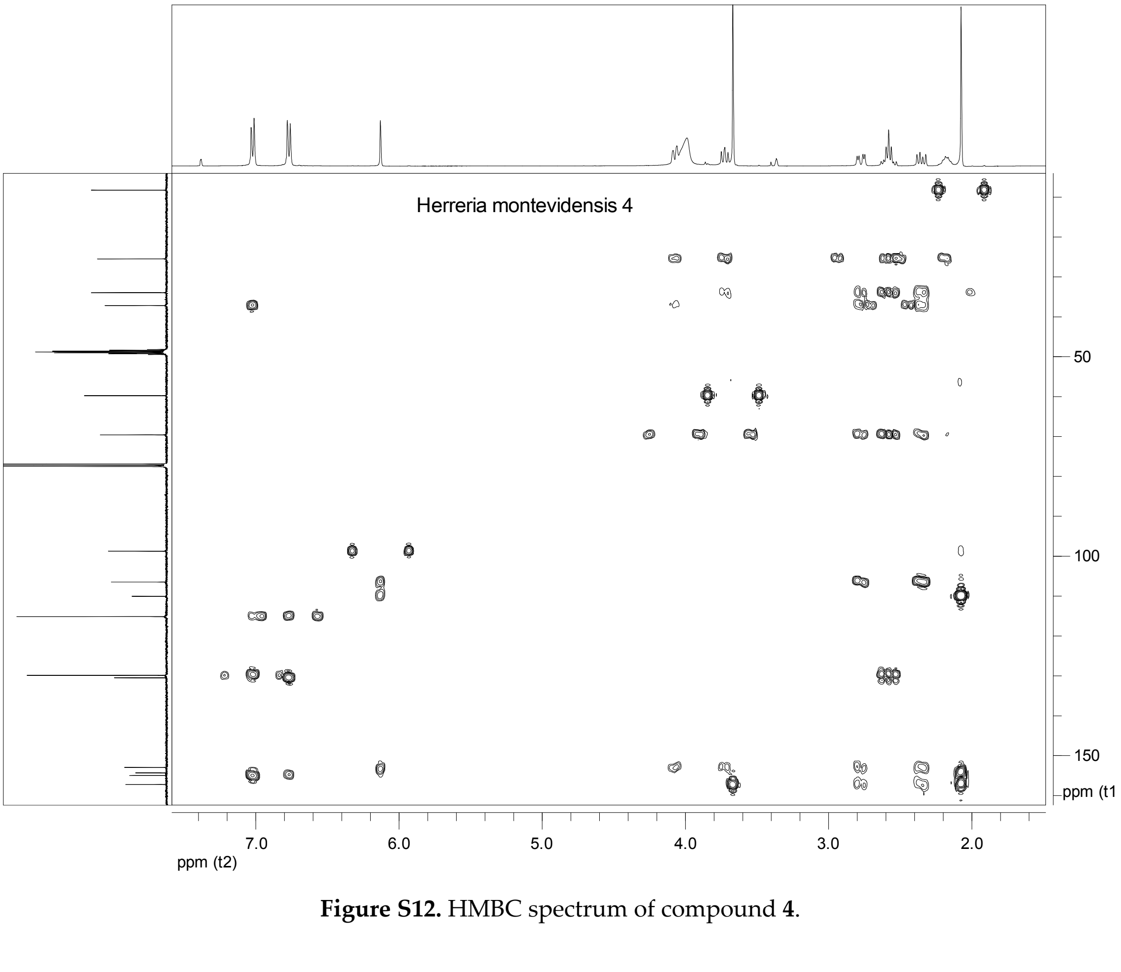

Figure S12. HMBC spectrum of compound **4**.

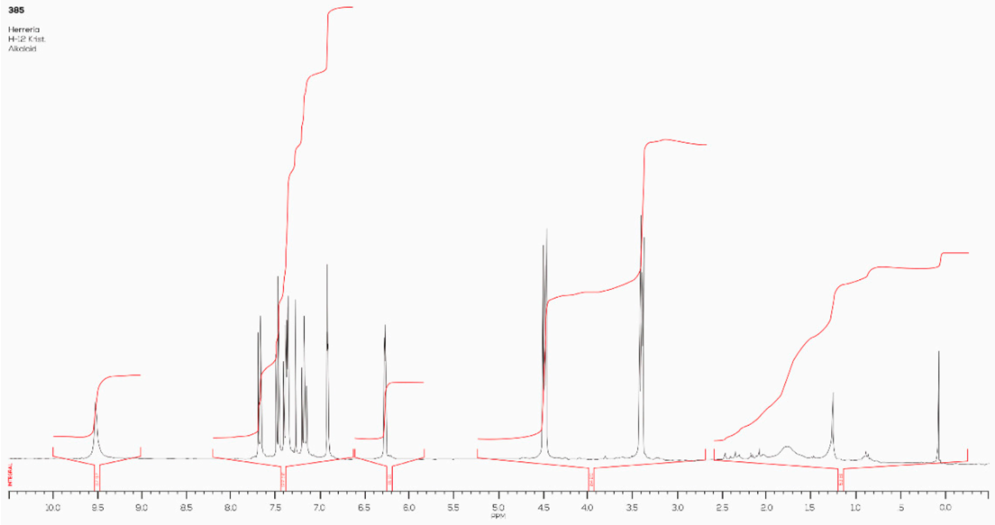

Figure S13. ^{1}H-NMR (400 MHz, $CDCl_3$-MeOH-*d*$_4$) spectrum of compound **9**.

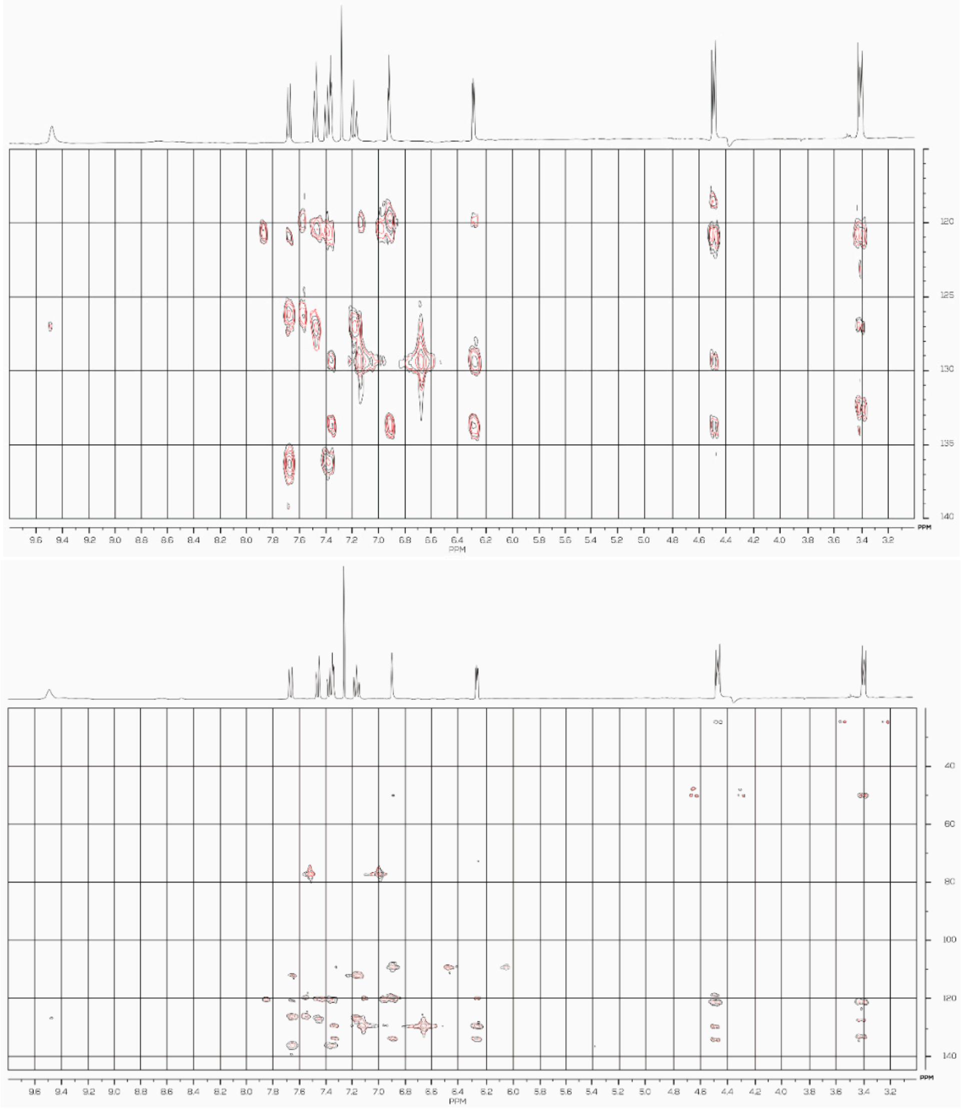

Figure S14. HMBC spectrum of compound **9**.
